# Chemical Constituents of Stinging Nettle (*Urtica dioica* L.): A Comprehensive Review on Phenolic and Polyphenolic Compounds and Their Bioactivity

**DOI:** 10.3390/ijms25063430

**Published:** 2024-03-18

**Authors:** Saša Đurović, Ivan Kojić, Danka Radić, Yulia A. Smyatskaya, Julia G. Bazarnova, Snežana Filip, Tomislav Tosti

**Affiliations:** 1 Institute of General and Physical Chemistry, Studentski trg 12/V, 11158 Belgrade, Serbia; sasatfns@uns.ac.rs (S.Đ.); danka.radic81@gmail.com (D.R.); 2 Graduate School of Biotechnology and Food Industries, Peter the Great Saint-Petersburg Polytechnic University, Polytechnicheskaya Street, 29, 195251 Saint-Petersburg, Russia; smyatskaya_yua@spbstu.ru (Y.A.S.); jbazarnova@spbstu.ru (J.G.B.); 3 Innovative Centre Faculty of Chemistry Belgrade, University of Belgrade, Studentski trg 12-16, 11158 Belgrade, Serbia; ivankojic@chem.bg.ac.rs; 4 Technical Faculty “Mihajlo Pupin” Zrenjanin, University of Novi Sad, Djure Djakovica b.b., 23000 Zrenjanin, Serbia; filipsnezana@gmail.com; 5 Institute of Chemistry, Technology and Metallurgy—National Institute of the Republic of Serbia, University of Belgrade, Njegoševa 12, 11000 Belgrade, Serbia

**Keywords:** stinging nettle, phenolic acids, polyphenolic compounds, chemical profile, biological activity, mechanisms of action

## Abstract

Polyphenolic compounds are of great interest in today’s science. Naturally, they occur in plants and other sources in many different forms. Their wide range of biological activity has attracted the attention of the scientific community. One of the sources of phenolic compounds is stinging nettle (*Urtica dioica* L.), a common plant in almost all parts of the world. A long tradition of utilization and an interesting chemical profile make this plant a fascinating and extensive object of study. The chemical profile also allows this plant to be used as a food and a pigment source in the food, pharmaceutical, and cosmetic industries. Previously conducted studies found phenolic acids and polyphenolic compounds in root, stalk, and stinging nettle leaves. Different extraction techniques were usually used to isolate them from the leaves. Obtained extracts were used to investigate biological activity further or formulate different functional food products. This study aimed to collect all available knowledge about this plant, its chemical composition, and biological activity and to summarize this knowledge with particular attention to polyphenolic compounds and the activity and mechanisms of their actions.

## 1. Introduction

Consumption of plants and their products has significantly increased over the last several decades due to the expense of synthetic products, their undesired effects on human health, and the constantly growing awareness of the advantages of plant consumption [1]. Studies showed that plants contain many compounds responsible for their beneficial and nutritional values. Those compounds have numerous biological activities such as antioxidant, antimicrobial, cytotoxic, anti-inflammatory, antiviral, and many other beneficial effects for human health [2,3]. Moreover, the consumption of plants as food is connected to reduced risks of different diseases and disorders [4]. All these effects of the plants are usually related to phenolic compounds, which are synthesized by the plants as part of secondary metabolism [5].

To answer the increasing demand for healthier food products, developing new approaches for isolating biologically active compounds from their natural sources and their utilization in functional products is becoming increasingly significant. Different extraction techniques have been developed during the second half of the 20th century. Even dough, development, and improvement of green techniques are still in progress. The modern trend is the application of nonconventional extraction techniques using environmentally friendly solvents such as ethanol or water [6]. Soxhlet extraction is a conventional technique considered standard for comparing the performance with other extraction techniques. However, it usually goes with applying toxic organic solvents, which is the main drawback [7]. To overcome drawbacks, nonconventional techniques have been developed to isolate polyphenolic and other natural compounds from their natural sources [5]. Those techniques are ultrasound-assisted, microwave-assisted, subcritical water, and supercritical fluid extraction, allowing the application of non-toxic, cheap, and environmentally friendly solvents [5,6,8,9].

**Figure 1:**
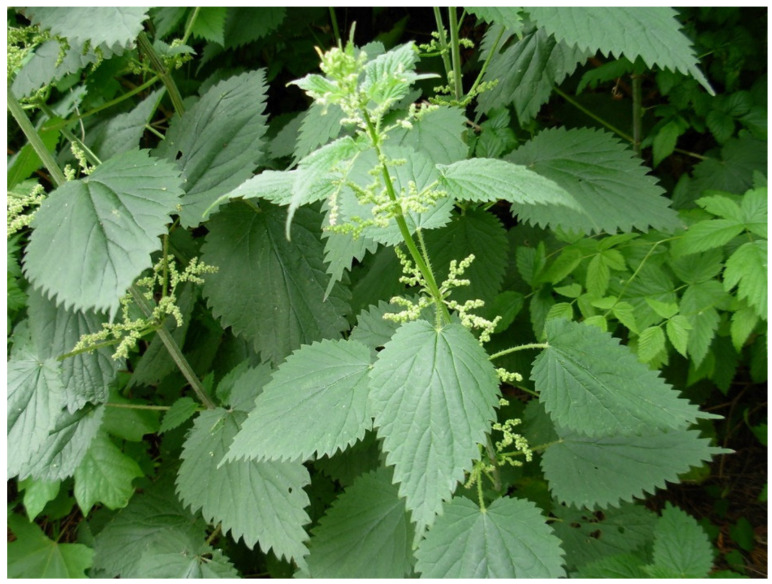
Stinging nettle (photo taken by Tomislav Tosti).

Stinging nettle (Figure 1) is a widely known plant for its application in folk medicine, pharmacy, cosmetics, the food industry, and as a part of many dishes [10,11,12]. Moreover, this plant is also cultivated for commercial green pigment chlorophyll, also known as E140 [13]. The binomial name of this plant is *Urtica dioica* L., and it belongs to the *Urticaceae* botanical family [14].

The plant is widely spread globally but is common in Europe, North America, North Africa, and some parts of Asia [12]. Medicinal applications consider fresh leaves and extracts for treating flailing arthritic or paralytic limbs, stimulating blood circulation, and warmth of joints and extremities [15]. The stinging nettle’s extracts showed different biological activities such as antioxidant, antimicrobial, anti-inflammatory, anti-ulcer, and analgesic [16,17,18,19,20,21,22,23]. This plant is also used as a medicament against anemia, gout, eczema, and urinary, bladder, and kidney problems [14,24,25,26]. Stinging nettle is also part of many dishes such as soups, omelets, noodles, salads [10], and different functional food products like bread [27], cakes and cookies [28,29], chocolates [30], drinks and beverages [31,32,33], edible oils [34,35], dairy products [36,37], meat and fish products [38,39,40,41], and many others. Another application of this extraordinary plant is as fertilizer such as slurry, which has become increasingly popular in Spain [12].

The whole plant’s full potential remains unknown; further studies are necessary to expand our knowledge about this plant and its value. So far, we have discovered that it contains a lot of compounds valuable for human nutrition and health. Bearing in mind that stinging nettle has been used for centuries as medicine and for food, this review article aims to present up-to-date knowledge about the chemical profile of stinging nettle, focusing on polyphenolic compounds as one of the leading carriers of this plant’s activity. Besides the profile, the bioactivities of the phenolic and polyphenolic compounds are also presented and discussed in the following sections.

## 2. Chemical Profile of Stinging Nettle

Today, it is clear that the biological activity of plants originates from their chemical composition. A wide range of natural compounds are responsible for the activity, but their interaction enhances it, which is known as synergy [5,8,9]. Understanding the significance of chemical profiles forced scientists to deal more thoroughly with applying different analytical techniques and methods to analyze natural compounds in their sources and extracts. For the same reason, many modern analytical methods were used in the chemical characterization of stinging nettle [11].

Chemical analysis of stinging nettle showed the presence of different classes of chemical compounds that are considered beneficial for human health. Among them are terpenes, metals, vitamins, fatty acids, carotenes, polyphenolic compounds, amino acids, and many others [14,23,26,42,43,44,45,46,47,48]. However, the first and the most complete study of the chemical profile of stinging nettle was reported by Đurović et al., where terpenes, phenolic acids, polyphenols, chlorophyll, carotenoids, fatty acids, and minerals were analyzed [11]. Of terpenes, 12 compounds were found: limonene, α-pinene, β-pinene, γ-terpinene, linalool, geraniol, camphor, eucalyptol, α-terpineol, carvacrol, eugenol, methyl chavicol. Linalool was the principal compound, followed by carvacrol and eugenol [11]. Although the presence of these three compounds was previously reported in relatively high amounts, carvacrol was reported to be the principal compound [42], confirming the influence of geographical origin on the chemical profile.

Carotenoids and chlorophylls A and B were also reported in stinging nettle. Đurović et al. performed the Soxhlet extraction technique to isolate these pigments using different solvents. Results showed that 96% ethanol was the best solvent for extracting chlorophylls, while methylene chloride was the best for carotenoids. However, this solvent showed excellent results in isolation [11]. Moreover, supercritical fluid extraction (SFE) proved to be an efficient technique for isolating chlorophylls and carotenoids [18]. However, it should be noted that the SFE’s effectiveness strictly depends on extraction conditions, i.e., pressure and temperature [18]. Besides total carotenoid content, which other research groups reported [44,49], individual carotenoids were identified and their contents were reported. Thus, β-carotene, neoxanthin, violaxanthin, lutein, and lycopene were reported in stinging nettle [43,44]. Chlorophyll content was also reported by Kukrić et al. [23], who also reported total carotenoid content. Reported results showed diversity in the contents of these pigments, which could be ascribed to geographical and seasonal diversities in plant development and, therefore, in the synthesis of these pigments.

Fatty acids are another class of compounds found in stinging nettle. Studies showed that C18:3 was the principal fatty acid in stinging nettle leaves, followed by C18:2 and C16:0 [45]. Guil-Guerrero investigated the content of fatty acids in young and mature leaves. C18:3 was the principal fatty acid in both cases, but its content significantly differed. C18:3 content in mature leaves was 40.7%, while in young leaves was only 29.6% [43]. Đurović et al. investigated the fatty acid profile and content in stinging nettle leaves using different extraction techniques and solvents. The fatty acid profile in different parts of the plant reported by various research groups is shown in Table 1.

Results in Table 1 indicate a strong influence of both extraction technique and applied solvent on the chemical profile. When petroleum ether was used, 79.77% were saturated fatty acids and 20.23% unsaturated fatty acids. C12:0 was the principal fatty acid in the isolated residue, followed by C16:0 and C18:1 [51]. On the other hand, the classic extraction technique with a formaldehyde-ethanol mixture gave an entirely different profile of isolated residue. In this case, unsaturated fatty acids were slightly efficiently isolated (52.70%), with C18:3 as the primary compound, followed by C16:0 and C18:2 [11]. Besides the previously mentioned differences, the absence of small-chain fatty acids might be noticed in the residue obtained by the classic technique with the formaldehyde-ethanol mixture (Table 1).

Furthermore, Đurović et al. also investigated the influence of the operational parameters (pressure and temperature) of SFE on the fatty acid profile in obtained extracts [18]. Results showed that mild pressure (200 bar) and elevated temperature (60 °C) are the best parameters for isolating saturated fatty acids and obtaining the highest yield. However, unsaturated fatty acids (both mono and polyunsaturated fatty acids) were extracted more efficiently at lower pressure (100 bar) and temperature (40 °C) [18]. The most recent study showed that unsaturated fatty acids were the most abundant, especially C18:2, in the leaves and roots of the stinging nettle. Interestingly, the high content of both *cis*- and *trans*-C18:1 was also reported together with the high content of C16:1 [50]. The content of saturated fatty acids was relatively low, which is quite interesting when considering that the authors used Soxhlet extraction and petrol ether as a solvent.

The content of metals and minerals is also one of the quality parameters considered very important. The presence of bulk elements (K, Na, Ca, and Mg) and some trace elements (e.g., Fe and Zn) is significant from the application of this plant as a food. It is well-known that stinging nettle is rich in iron, which was confirmed in different studies [15,47,48]. Đurović et al. [11] and Popov et al. [54] investigated the content of metals and minerals in stinging nettle leaves and their extracts were obtained using different extraction techniques. Besides the high content of bulk elements, elevated Fe content was confirmed in wild-growing Serbian stinging nettle (Table 2).

Popov et al. investigated metal and mineral content in stinging nettle leaf extracts prepared using Soxhlet extraction, maceration, ultrasound-assisted, and microwave-assisted extraction techniques [54]. Results confirmed a high content of bulk elements and Fe. However, only a tiny portion of Fe was transferred into the extracts during extraction (3.53–8.24 mg/L) [54]. Therefore, if it is the goal to use this plant as a source of minerals, then the best way is to ingest it directly.

The next class of chemical compounds of interest to the scientific community is vitamins. In this case, it was reported that stinging nettle is rich in vitamin C and vitamin B series [15]. The content of these compounds was studied after extracting them from stinging nettle leaves using Soxhlet extraction (SE), maceration (MAC), ultrasound-assisted UAE), and microwave-assisted extraction (MAE) techniques [16]. Vitamins in stinging nettle leaves and their extracts are summarized in Table 3.

Results showed that UAE and MAE were generally the best choice for isolating the vitamins from the plant’s leaves. Different results between these two techniques may be ascribed to different mechanisms of extraction as well as the stability of desired compounds. The highest difference I might have noticed was in the case of vitamin B_6_ content, which was 104.15 mg/L in UAE and only 7.14 mg/L in MAE extract. On the contrary, vitamin B_3_ was extracted more efficiently by MAE (197.35 mg/L) than by UAE (28.37 mg/L).

Besides fatty acids, vitamins, minerals, and metals, the content of other classes such as tannins, carbohydrates, sterols, polysaccharides, isolectins, and amino acids were also reported to be found in stinging nettle [15,23,43,46].

## 3. Phenolic and Polyphenolic Compounds in Stinging Nettle

Due to their high biological potency, phenolic and polyphenolic compounds are considered one of the most significant classes of organic compounds [57,58]. They are products of the secondary metabolism in plants. There are several groups of these compounds, i.e., simple phenolics, phenolic acids, stilbenes, flavonoids, coumarins, tannins, lignans, and others [5,59] or their derivates or isomers [58]. They can be found only in conjugated form with carbohydrates linked to hydroxyl or aromatic carbon. Moreover, conjugation with other structural forms such as amines, carboxylic and other organic acids, lipids, and other phenols is also identified [60]. Natural or dietary polyphenols may be commonly found in fruits, vegetables, plants, cereals, and microalgae, and they are ascribed to a wide range of biological activities, making them popular in the science and food industry as well [57,58].

Both phenolic and polyphenolic compounds are also found in stinging nettle. There are many different studies regarding the presence and content of these compounds in stinging nettle. However, these results differ due to several reasons, e.g., geographical, seasonal, and environmental variations and conditions, part of the plant used for extraction (root, stalk, leaves), extraction technique, and solvent or mixture of solvents used for the extraction [11,16,17,19,61,62,63,64]. Đurović et al. reported a polyphenolic profile of stinging nettle leaves obtained using different extraction techniques and 96% ethanol as a solvent [11]. The two prepared extracts mainly differed in the presence/absence of the phenolic acids. Otles and Yalcin analyzed phenolic compounds in root, stalk, and leaves. The authors reported the lack of gallic acid and catechin in analyzed parts of the plant [63], which is in agreement with other studies [16,17] but contrary to other studies that reported the presence of only gallic acid [11,64] or both compounds [19,61]. The results showed that leaves were the richest in polyphenolic compounds, followed by stalk and root [63]. Compounds like syringic acid, myricetin, quercetin, kaempferol, rutin, ellagic acid, isorhamnetin, *p*-coumaric acid, ferulic acid, and naringin were found and quantified in all samples [63]. Besides the compounds mentioned above, other phenolic acids and polyphenolic compounds were also reported in leaves but with a specific diversity because of the extraction techniques used (Table 4).

In this case, both glycosides and aglycones were reported in extracts. The presence of aglycones indicates hydrolysis during the extraction processes. Moreover, compounds like gentisic acid, umbelliferone, scopoletin, chrysoeriol, and genistein were reported in addition to the previous studies [19]. Other glycosidic forms such as kaempferol-pentoside, kaempferol-rhamnoside, quercetin-acetyl-rutinoside, and kaempferol-pentosyl-hexoside were also reported to be isolated from stinging nettle leaves [61,64]. Đurović et al. also reported the presence of taxifolin, genkwanin, acacetin, chrysin, chrysoeriol, eriodictyol, and daidzin [16]. Interestingly, all these compounds, except taxifolin and daidzein, were found and quantified only in the Soxhlet extract of stinging nettle leaves. Further extraction of exhausted leaves after supercritical fluid extraction also showed the dependence of the chemical profile on the technique [17]. The authors compared the polyphenolic profile of extracts obtained from the dried plant material and plant material after the supercritical fluid extraction (SFE). Polyphenolic compounds were extracted using ultrasound-assisted (UAE) and microwave-assisted (MAE) extraction techniques. Reported results revealed the influence of UAE and MAE techniques on the polyphenolic profile but also showed that SFE conditions (pressure and temperature) significantly impacted the prepared extracts’ polyphenolic profile. The MAE was a more efficient technique for extracting phenolic compounds than the UAE, and more important was the fact that there was a significant loss of phenolic and polyphenolic compounds after the SFE. The total content of quantified compounds decreased more than double in the UAE and was even sharper in the MAE, indicating that some of these compounds were extracted during the SFE. Possible interactions and reactions during the UAE and MAE could impact the final result [16].

## 4. Biological Activity of Phenolic and Polyphenolic Compounds

**Figure 2:**
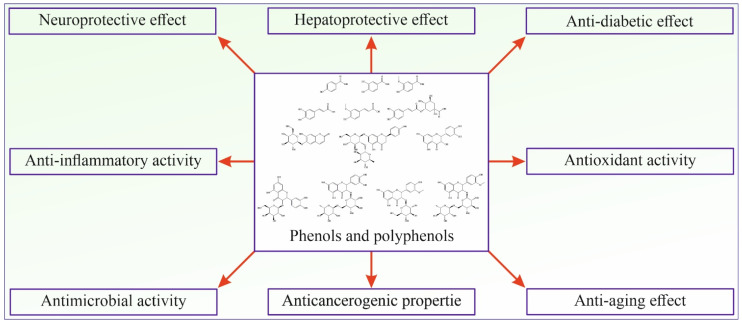
Schematic presentation of phenolic and polyphenolic bioactivities.

Phenolic and polyphenolic compounds show many different activities beneficial for human health and are considered one of the most important classes of natural compounds (Figure 2).

### 4.1. Antioxidant Activity

The antioxidant activity of phenolic and polyphenolic compounds has been and still is one of the most studied properties. Many spectrophotometric tests have been developed for in vitro investigation of antioxidant activity and are still widely applied [57]. The in vivo study showed that polyphenols bind to lipids in blood and reduce lipid peroxidation [68]. Dihydrocaffeic acid scavenges superoxide anions, hydroxyl, and peroxyl radicals in human endothelial cells [69]. Quercetin and curcumin increase glutathione peroxidase activity, superoxide dismutase, catalase, and glutathione reductase in vitro and in vivo [70,71,72]. Generally, it is considered that these compounds’ antioxidant activity mechanisms are H atom transfer, transfer of electrons, and chelation ability of metal ions [73,74]. Transfer of the H atom is more favorable than electron transfer because higher energy is needed for the electron transfer process. On the other hand, free hydroxyl groups allow polyphenolic compounds to interact with transition metals and chelate them [73].

### 4.2. Cardioprotective Activity

It has been shown that polyphenolic compounds can decrease the risk of atherosclerosis and exert cardioprotective activity [57,58]. Studies showed that the intake of polyphenols reduces the risk of thrombosis [75,76,77]. Oleuropein inhibits the oxidation of LDL (low-density lipoprotein) in in vitro tests [78]. At the same time, quercetin regulates the expression of serum HDL-associated paraoxonase 1 [79], inhibits oxidized LDL-triggered apoptosis, and increases intracellular glutathione regulation [80]. Proanthocyanidin appears to reduce cardiomyocyte apoptosis through the inhibition of ischemia/reperfusion-induced activation of JNK-1 and c-Jun [81]. It also regulates CD36 mRNA and protein levels in oxidized LDL-treated peripheral blood mononuclear cells [82]. Resveratrol decreases the expression of vascular cell adhesion molecule-1 [83], cyclooxygenase-2 [84], and matrix metalloproteinase-9 mRNA [85], suppressing the nuclear factor AP-1 activation [84]. Intake of berries and red fruits could reduce cancer through several different mechanisms, such as inhibition of cytochrome P450-dependent monooxygenase 1A1, blockage of the epidermal growth factor receptor tyrosine kinase, and decreasing protein kinase CKII activity [58].

### 4.3. Anticancerogenic Properties

Polyphenols can interfere with the biochemical processes, interfering with carcinogenesis [58]. Anticancer effects have been noticed in the mouth, stomach, duodenum, colon, liver, lung, and skin [57]. To date, many different polyphenolic compounds have been tested and shown protective effects in specific models, but the mechanisms of their action are different [86]. In vitro tests showed inhibition of cellular proliferation and viability followed by apoptosis of 4T1 cancer cells after interacting with proanthocyanidins in a time- and dose-dependent manner [87]. Catechin, epigallocatechin-3-gallate, and epigallocatechin-gallate inhibit proteosome and induce tumor cell death [88]. Gallic acid inhibits damage to the DNA, preventing colon carcinogenesis. Intake of these compounds increases the level of antioxidants, enhances the activity of ascorbic acid, and elevates the level of α-tocopherol [89]. The core structure of flavones reduces the proliferation of HT-29 cancer cells and induces differentiation and apoptosis [90]. Curcumin inhibits cellular proliferation and angiogenesis in vitro and in vivo tests [91,92]. Resveratrol blocks activation of the MAPKs and AP-1 in mice skin [93].

### 4.4. Anti-Inflammatory Activity

Inflammation is considered to be a crucial factor in several diseases, such as obesity, type II diabetes, cardiovascular diseases, neurodegenerative diseases, and aging [57]. Oxidative stress induces inflammation mediated by activation of NF-κB and AP1 followed by expression of pro-inflammatory genes IL-1β, IL-8, tumor necrotic factor alpha (TNF-a), and inducible nitric oxide synthase (iNOS) [58]. A study of 18 polyphenolic metabolites derived from colon microbiota was conducted by screening prostaglandin E-2 production. Results showed that hydrocaffeic, dihydroxypheyl acetic, and hydroferulic acids successfully inhibited over 50% of prostaglandin E-2 synthesis. It was also noticed that hydrocaffeic acid successfully inhibited the expression of cytokines IL-1β, IL-8, and TNF-a, reduced MDA levels, and oxidative damage of DNA in distal colon mucosa [94]. It was also reported that oleuropein inhibited IL-1β production by 80%, and caffeic acid performed the same by 40% at a concentration of 10^−4^ M. Kaempferol diminished the level of prostaglandin E-2 by 90% at the same concentration [95]. Resveratrol inhibits the expression of pro-inflammatory genes, inhibiting the inhibitory κB (IκB), followed by the inhibition of NF-κB transactivation, and activates deacetylases [96]. Apigenin, luteolin, and quercetin inhibit inflammatory responses through downregulation of the iNOS and adhesion molecule expression in NR8383 and human endothelial cells [97,98,99].

### 4.5. Antimicrobial Activity

The antimicrobial activity of polyphenolic compounds is widely known and still being investigated. They showed activity against bacteria, fungi, and viruses [57]. Conducted studies showed activity against different microbes [5,8,9]. They act through different mechanisms, including forming hydrogen bonds, covalent bonds, and hydrophobic effects, resulting in the inactivation of microbial adhesins, enzymes, cell envelope transport proteins, and others [100]. It was reported that apigenin and quercetin inhibit DNA gyrase in *E. coli* [101], and naringenin changes fluidity in inner and outer membrane layers [102]. Studying the structure–activity relationship indicated the importance of the hydroxyl group for antimicrobial activity [103]. Pinosylvin, pinosylvin monomethyl ether, and piceatannol also showed antimicrobial activity, destabilizing the outer membrane of Gram-negative bacteria and interacting with the membrane itself [104]. Polyphenolic compounds can also suppress numerous microbial virulence factors, e.g., host ligand adhesion reduction, biofilm formation inhibition, bacterial toxin neutralization, and synergistic effects with antibiotics [105]. Moreover, it has been found that galanin reverses bacterial resistance to β-lactam types of antibiotics in the case of S. aureus [106].

### 4.6. Other Biological Activities of Polyphenolic Compounds

In addition to the abovementioned and described activities, phenolic and polyphenolic compounds showed many other biological activities. Thus, they expressed anti-aging effects, neuroprotective effects, positive effects on digestive enzymes and maintenance of gastrointestinal health, modulation of signal transduction pathways, improvement of endothelial functions, protective effects on the function of immune cells, antiallergic activity, anti-diabetic effects, regulation of the cell cycle progression, modulation of hormonal effects and contraceptive activity, and effects in the treatment of chronic obstructive pulmonary disease [57,58]. However, the full potential of these compounds still needs to be fully revealed and further studies are necessary. They are in progress, providing new results and insight into the effects and mechanisms of activity.

## 5. Bioactivity of Phenolic and Polyphenolic Compounds in Stinging Nettle

### 5.1. Bioactivity of Most Abundant Phenolic and Polyphenolic Acids in Stinging Nettle

**Figure 3:**
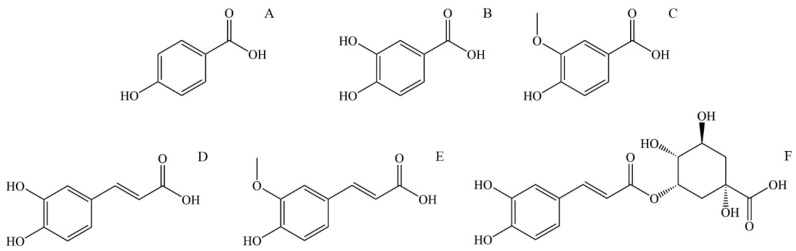
The structural formula of most abundant phenolic and polyphenolic acids in stinging nettle (*Urtica dioica*): (**A**) *p*-Hydroxybenzoic acid, (**B**) Protocatechuic acid, (**C**) Vanillic acid, (**D**) Caffeic acid, (**E**) Ferulic acid, (**F**) 5-*O*-Caffeoylquinic acid.

*p*-Hydroxybenzoic acid (4-Hydroxybenzoic acid, Figure 3A) is widely distributed in plants. It is shown that it has antioxidant, antimicrobial, and estrogenic activity. Like all polyphenolic acids, p-hydroxybenzoic acid has antioxidant activity and acts as ROS (reactive oxygen species) and is a free radical scavenger. *p*-Hydroxybenzoic acid shows an antimicrobial effect on most Gram-positive and some Gram-negative bacteria. The limiting factor of its microbial effect is polarity, so it is shown that alkyl esters of *p*-hydroxybenzoic acid, better known as parabens, show increased microbial activity with increased length of the *n*-alkyl chain [107]. *p*-Hydroxybenzoic acid and especially its alkyl esters (parabens) express estrogenic activity in vivo due to their similarity to 17-β-estradiol. Although their estrogenic activity is 1000–1,000,000 times lower than estradiol, parabens are of interest mainly because they are used as preservatives in cosmetic products (usually 0.1% and sometimes 0.5%) [108]. Due to their increased hydrophobic properties, parabens are easily absorbed through the skin and are metabolized in the liver to form p-hydroxybenzoic acid [107].

Protocatechuic acid (3,4-Dihydroxybenzoic acid, Figure 3B) is one of the most commonly found polyphenolic acids widely distributed in various foods, and thus, it is a standard part of the human diet. Some bioactive properties are antimicrobial, antioxidant, anti-inflammatory, anti-carcinogenic, anti-diabetic, and cardio, neuro, and nephronprotective [109]. It has been found that protocatechuic acid expresses an antimicrobial effect on certain bacteria responsible for spoiling food. Further research conducted by microdilution assay showed an antibacterial effect on Gram-negative and Gram-positive bacteria, mainly due to growth inhibition by bacteria membrane lysis [110]. Compounds that show an antioxidant effect can directly or indirectly scavenge reactive oxygen species (ROS) or free radicals generated as a metabolism product. Metabolism imbalance can lead to a buildup of ROS and free radicals and causes damage to surrounding tissue by triggering apoptosis, adding to the pathogenesis of cardiovascular and neurodegenerative diseases, diabetes, cancer, and other diseases. Protocatechuic acid shows an antioxidant effect by increasing the activity of glutathione peroxidase and superoxide dismutase. Also, protocatechuic acid is an effective peroxyl radical scavenger in an aqueous environment [111]. In an in vivo study on mice with lipopolysaccharide-induced lung injury, protocatechuic acid showed an anti-inflammatory effect by inhibiting inflammatory cytokine tumor necrosis factor α, interleukin-1β, and interleukin-6 [109]. The neuroprotective effect of protocatechuic acid was studied in the culture of cortical brain cells and it was found that it promotes cell growth by neutralizing the effect of H_2_O_2_. Also, the activating effect on endogenous antioxidant enzymes is an essential effect of protocatechuic acid that can lead to improved brain recovery and even be applied to treating neurodivergent diseases [112]. The anti-carcinogenic effect of protocatechuic acid is mainly expressed by its antioxidant abilities, scavenging ROS, and preventing damage to the mitochondrial membrane, thus preventing cell apoptosis.

Vanillic acid (4-Hydroxy-3-methoxybenzoic acid, Figure 3C) is a natural phenolic acid detected in many plants. It has antioxidant, antimicrobial, anti-carcinogenic, neuroprotective, anti-inflammatory, anti-obesity, cardioprotective, and hepatoprotective properties. Regarding anti-carcinogenic activity, vanillic acid shows cytotoxic and anti-proliferative activity on several kinds of carcinoma. Vanillic acid induces mitochondrial-influenced apoptosis and DNA damage of lung cancer cells. Additionally, vanillic acid inhibits the synthesis of hypoxia-induced factor-1α, expression of the hypoxia-induced protein, and transcriptional activation in human colon cancer cell culture [113]. The neuroprotective activity of vanillic acid is expressed by its antioxidant properties, mainly through protection against lipid peroxidation and protein oxidation and in affecting antioxidant enzymes like glutathione, catalase, and superoxide dismutase. The neuroprotective effect is also expressed by vanillic acid anti-inflammatory activity inhibiting the interleukin-1β, interleukin-6, and tumor necrosis factor α (TNF-α) protein expression [114]. The anti-obesity effect of vanillic acid is demonstrated by its anti-inflammatory activity. Additionally, vanillic acid can increase glucose-induced insulin release [113]. Vanillic acid expresses antimicrobial activity by causing microbe membrane degradation by reducing ATP concentration, pH, and membrane potential in microbial cells [115].

Caffeic acid (3,4-dihydroxycinnamic acid, Figure 3D) is present in many vegetables such as potatoes, carrots, olives, bat, coffee, and propolis. It can be found as a monomer, a derivate (such as ester, amide, or glycoside), a dimer, a trimer, or a derivate with flavonoids. The primary use of caffeic acid in plants is to protect against pests but also for UV protection [116]. It is known that caffeic acid has antioxidant, antimicrobial, cardioprotective, anti-diabetic, hepatoprotective, and anti-carcinogenic activity. Caffeic acid is a potent antioxidant and a primary and secondary antioxidant. During the primary antioxidant activity, caffeic acid reacts with generated free radicals by donating electrons or hydrogen, thus neutralizing them to stable products. As a secondary antioxidant, it can act as a chelating agent, forming complexes with iron and copper, preventing peroxide decomposition and reducing the generation of free radicals [117]. This secondary antioxidant ability is also helpful in cancer treatment since the chelate of caffeic acid with copper has a pro-oxidant ability to induce lipid peroxidation that can damage cancer cells by generating covalent adducts with cancer DNA [118]. The antioxidant property of caffeic acid and its derivates is also a source of antimicrobial and cardioprotective activity. The concentration of oxidized low-density lipoprotein (LDL) in serum significantly influences the generation of cardiovascular diseases such as atherosclerosis. The origin of oxidized LDL through the reaction of oxygen free radicals with LDL particles is influenced by copper (II) ions. Caffeic acid lowers the oxidation of LDL by reducing the consumption of vitamin E and the generation of malondialdehyde and lipofuscin [119]. Assays with diabetic mice supplemented with caffeic acid showed a cardioprotective effect and improved lipid metabolism and glycemic control [120]. Caffeic acid has also decreased glucose levels in insulin-resistant rats after intravenous application of caffeic acid. Following that treatment, rats have shown reduced plasma glucose levels during glucose tolerance tests [121].

Ferulic acid (4-hydroxy-3-methoxycinnamic acid, Figure 3E) is a derivative of caffeic acid, and similar to caffeic acid, it has antioxidant properties, mainly due to a phenolic ring with a conjugated side chain. Apart from that, ferulic acid has antimicrobial, anti-diabetic, anti-inflammatory, anti-thrombotic, vasodilatory, hepatoprotective, and anti-carcinogenic effects. As mentioned, ferulic acid is an effective antioxidant with ROS-scavenging properties, protecting DNA and lipid molecules from oxidation and preventing cell damage [122]. Commonly observed effects of diabetes are elevated glucose levels (hyperglycemia), increased generation of free radicals, and oxidative stress. Oxidative stress causes an imbalance in the homeostasis of antioxidants and pro-oxidants, leading to cell damage [123]. The antioxidant effect of ferulic acid lowers the plasma glucose level by stimulating insulin production and prevents damage to pancreas cells by its antioxidant effect. Ferulic acid can prevent free radical generation in leucocytes induced by nicotine, preventing its effect on lipid oxidation and lowering glutathione levels. It is also shown that ferulic acid inhibits the growth of cancer cells [124].

5-*O*-Caffeoylquinic acid (4-*O*-(3,4-Dihydroxycinnamoyl)-quinic acid, Figure 3F) is widely spread in plants, particularly medical plants. 5-*O*-Caffeoylquinic acid has antioxidant, anti-inflammatory, neuroprotective, cardioprotective, and anti-diabetic effects. Similar to other phenolic and polyphenolic acids, the presence of a phenolic core conjugated with the *n*-alkane chain is the source of its antioxidant properties by in vivo oxidation to quinoids [125]. Thus, 5-*O*-caffeoylquinic acid is an effective free radical and ROS scavenger with good antioxidant and anti-inflammatory effects. Neuroprotective activity is also expressed by protection from oxidative stress and by normalizing calcium homeostasis [126]. Caffeoylquinic acid can potentially reduce the risk of type 2 diabetes development by activating adenosine monophosphate kinase [125].

### 5.2. Bioactivity of Most Abundant Coumarins and Flavonols in Stinging Nettle

**Figure 4:**
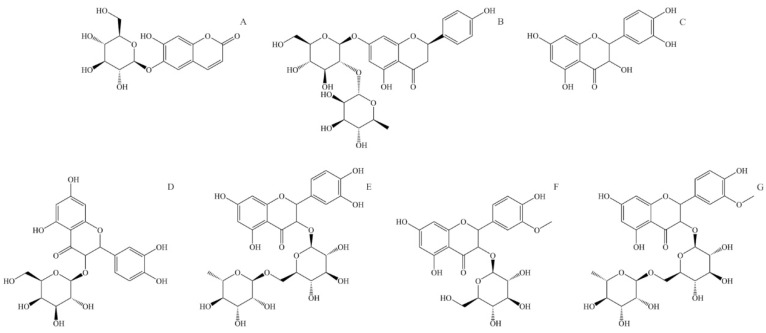
Structural formulas of most abundant flavonoids in stinging nettle (*Urtica dioica*). (**A**) Aesculin, (**B**) Naringin, (**C**) Quercetin, (**D**) Quercetin-3-*O*-galactoside, (**E**) Rutin (quercetin 3-*O*-rutinoside), (**F**) Isorhamnetin-3-O-glucoside, (**G**) Isorhamnetin-3-*O*-rutinoside.

Aesculin, also known as esculin (6,7-Dihydroxycoumarin-6-β-D-glucoside, Figure 4A), is a component of many well-known medical herbs. The biological effects of aesculin in vivo and in vitro are antibacterial, antioxidant, anti-inflammatory, anti-carcinogenic, and anti-arteriosclerotic. Aesculin is an effective free radical scavenger. It is found to improve the activity of superoxide dismutase and glutathione and reduce dopamine-dependent ROS overproduction in human neuroblastoma cells [127]. Aesculin is a potent anti-inflammatory compound and can suppress inflammatory factor expression like inducible nitric oxide synthase, interleukine-1β, and tumor necrosis factor-α [128]. Aesculin inhibits the proliferation of carcinoma cells through the mitochondrial apoptosis pathway and tumor cell inhibition [127]. Aesculin can also have an anti-arteriosclerotic effect by reducing triglyceride blood levels and inhibiting the proliferation of vascular soft muscle [129].

Naringin (4′,5,7-Trihydroxyflavanone-7-rhamnoglucoside, Figure 4B) is a naturally occurring flavone glycoside consisting of 4′,5,7-hydroxyflavone (naringenin) and rhamnose-β-1,2-glucose. Naringin has antioxidant, anti-inflammatory, anti-osteoporotic, and anti-carcinogenic properties; apart from that, it enhances the absorption of other medicaments, showing potential application in the pharmaceutical industry [130]. In human metabolism, naringin is metabolized in the liver through two-step hydrolysis, producing rhamnose and pruning in the first step and naringenin and glucose in the second. Naringin has relatively low bioavailability due to its low solubility and permeability. It is mainly absorbed in the intestine and is transformed by intestinal microflora to naringenin [131]. Naringin has a potent anti-inflammatory effect and acts as a very effective ROS and free radical scavenger. It also suppresses the production of inflammatory factors like interleukine-6, nitric oxide, nitric oxide synthesis, and tumor necrosis factor α [132]. Naringin shows in vivo tumor growth delay, increases phosphorylation of AMP-activated protein kinase, and inhibits cell growth; thus, it has the potential for treating specific cancer types [133].

Quercetin (3,3′,4′,5,7-pentahydroxyflavone, Figure 4C) is a widely distributed flavonoid in plants, and it can be found in a large number of fruits and vegetables. It is rarely found on its own but usually as a glycoside with different mono or disaccharides or in the form of esters or phenolic acids. The absorption rate of quercetin in the stomach and intestines depends on the form in which it is found. In stinging nettle, it can be found in free form but also as quercetin-3-*O*-galactoside (Figure 2D) and as rutin or quercetin 3-rutinoside (glycoside of quercetin and α-L-rhamnopyranosyl-(1→6)-β-D-glucopyranose) (Figure 2E). The bioactivity of quercetin is observed through antioxidant, anti-inflammatory, antimicrobial, and anti-carcinogenic effects [7]. Due to its low solubility and high polarity, quercetin has low bioavailability. The same can be said for quercetin glycosides, which are only slightly more water-soluble. It is shown that quercetin is an exceptionally effective ROS scavenger and a potent antioxidant [134]. Quercetin is also an effective anti-inflammatory compound, inhibiting inflammatory factors like tumor necrosis factor α and interleukine-1α [7]. Because of its antioxidant activity, quercetin can suppress the proliferation of cancer cells, mainly by reducing oxidative stress and suppressing multiple kinase proteins responsible for cancer cell growth [135].

Isorhamnetin or 3′-methoxyquercetin is a methoxylated derivate of quercetin (Figure 4D). Similar to quercetin, isorhamnetin is found bound to saccharides, and in stinging nettle, it is found as isorhamnetin-3-*O*-glucoside and isorhamnetin-3-*O*-rutinoside (glycoside of isorhamnetin and α-L-rhamnopyranosyl-(1→6)-β-D-glucopyranose). Isorhamnetin has antimicrobial, antioxidant, anti-inflammatory, and anti-carcinogenic effects [136]. Similarly to quercetin, isorhamnetin is an effective ROS and free radical scavenger. Isorhamnetin also expresses a strong anti-inflammatory effect and reduces pro-inflammatory cytokines like tumor necrosis factor α, interleukine-1β, and interleukine-6 [137].

## 6. Conclusions

Chemical profiling of stinging nettle showed that this plant contains many biologically significant compounds. The chemical profile of this plant explains its application during human history as a food source of pigments for food, pharmaceutical, and cosmetic industries. Phenolic compounds are one of the most exciting classes of natural products, extensively investigated due to a wide range of biological activities. Studies also proved that this plant contains them in aerial and underground parts. Because of their presence, many studies isolated them using conventional and nonconventional extraction techniques combined with different solvents. Prepared extracts were tested for different effects, e.g., antioxidant, antimicrobial, cytotoxic, and others. Furthermore, stinging nettle and its extracts were applied to formulate different functional food products and prepare many dishes. Studies also showed that the polyphenolic profile of extracts depends on the plant itself and the used extraction technique and solvent. Therefore, the preparation of the extract and further isolation of the polyphenolic compounds will depend on the application of the prepared material. However, despite progress in our knowledge about this plant, its chemical composition, and biological benefits, further studies are necessary to expand that knowledge and better utilize this amazing plant.

## Figures and Tables

**Table 1:** Fatty acid profile of stinging nettle.

Fatty Acid	Plant Part	Reference
Caproic acid (C6:0)	Leaf	[11,18,50,51]
	Root	[50]
Caprylic acid (C8:0)	Leaf	[11,18,50,51]
	Root	[50]
Capric acid (C10:0)	Leaf	[11,18,45,50,51]
	Root	[50]
Lauric acid (C12:0)	Leaf	[11,18,45,50,51,52,53]
	Root	[50]
Myristic acid (C14:0)	Leaf	[11,18,45,50,51,52]
	Root	[50]
Myristoleic acid (C14:1)	Leaf	[11,18,50,51,52]
	Root	[50]
Pentadecanoic acid (C15:0)	Leaf	[11,18,50,51]
	Root	[50]
*cis*-10-Pentadecenoic acid (C15:1)	Leaf	[11,18,51]
Palmitic acid (C16:0)	Leaf	[11,18,43,45,50,51,53]
	Stalk	[52]
	Root	[50,52]
Palmitoleic acid (C16:1)	Leaf	[11,18,43,45,50,51,52,53]
	Stalk	[52]
	Root	[50,52]
Heptadecanoic acid (C17:0)	Leaf	[11,18,50,51,52]
	Root	[50]
*cis*-10-Heptadecenoic acid (C17:1)	Leaf	[11,18,50,51,52]
	Root	[50]
Stearic acid (C18:0)	Leaf	[11,18,43,45,50,51,53]
	Stalk	[52]
	Root	[50,52]
*cis*-9-Oleic acid (C18:1n-9)	Leaf	[11,18,43,45,50,51]
	Stalk	[52]
	Root	[50,52]
*cis*-9,12-Linoleic acid (C18:2n-6)	Leaf	[11,18,43,45,50,51]
	Stalk	[52]
	Root	[50,52]
Arachidic acid (C20:0)	Leaf	[11,18,45,50,51,52,53]
	Root	[50]
*α-Linolenic acid* (C18:3 n-3)	Leaf	[11,18,43,45,50,51]
	Stalk	[52]
	Root	[50,52]
Heneicosanoic acid (C21:0)	Leaf	[11,18,50,51,52]
	Root	[50]
*cis*-11,14-Eicosadenoic acid (C20:2)	Leaf	[11,18,45,50,51,52]
Behenic acid (C22:0)	Leaf	[11,18,50,51,52,53]
	Root	[50]
Arachidonic acid (C20:4n-6)	Leaf	[11,18,45,50,51,52,53]
	Root	[50]
Tricosanoic acid (C23:0)	Leaf	[11,18,50,51,52,53]
	Root	[50]
*cis*-13,16-Docosadienoic acid (C22:2)	Leaf	[11,18,51,52]
Lignoceric acid (C24:0)	Leaf	[11,18,50,51,52,53]
	Root	[50]
*cis*-5,8,11,14,17-Eicosatrienoic acid (C20:5n-3)	Leaf	[11,18,50,51,52]
	Root	[50]
Nervonic acid (C24:1)	Leaf	[11,18,51,52]

**Table 2:** Elements and minerals in stinging nettle leaves.

Element	Plant Part	Reference
Bulk elements		
Na	Leaf	[11,47,54,55,56]
K	Leaf	[11,47,54,55,56]
Mg	Leaf	[11,47,54,55,56]
Ca	Leaf	[11,47,54,55,56]
Trace elements		
Fe	Leaf	[11,47,48,54,55,56]
Cu	Leaf	[11,47,48,54,55]
Ba	Leaf	[47]
Sr	Leaf	[47]
P	Leaf	[47]
Co	Leaf	[47,48]
Cr	Leaf	[47,48]
Ce	Leaf	[47]
La	Leaf	[47]
Mn	Leaf	[11,47,48,54,55]
Zn	Leaf	[11,47,48,54,55,56]
Cr	Leaf	[11,47,48,54]
Sn	Leaf	[11,54]
Ni	Leaf	[11,47,48,54]
Polluting elements		
Pb	Leaf	[11,48,54]
Cd	Leaf	[11,48,54]
Hg	Leaf	[11,54]
As	Leaf	[11,54]

**Table 3:** Vitamins C and B series in stinging nettle leaves extracts.

Vitamin	Plant Part	Reference
C	Leaf	[16,55]
B1	Leaf	[16,56]
B2	Leaf	[16,56]
B3	Leaf	[16,56]
B6	Leaf	[16,56]

**Table 4:** Polyphenolic profile of stinging nettle.

Compound	Plant Part	References
Protocatechuic acid	Leaf	[17,19,52,64,65,66]
	Stalk	[52,65]
	Root	[52,65]
*p*-Hydroxybenzoic acid	Leaf	[17,19,52,64,65,67]
	Stalk	[52,65]
	Root	[52,65]
Caffeic acid	Leaf	[16,17,52,53,64,65,67]
	Stalk	[52,63,65]
	Root	[52,65]
Vanillic acid	Leaf	[16,17,52,53,66]
	Stalk	[52,63]
	Root	[52]
Aesculin	Leaf	[16,17]
5-*O*-Caffeoylquinic acid	Leaf	[16,17,52,65]
	Stalk	[52,65]
	Root	[52,65]
*p*-Coumaric acid	Leaf	[16,17,64,65]
	Stalk	[65]
	Root	[65]
Ferulic acid	Leaf	[16,17,52,53,65,66,67]
	Stalk	[52,63,65]
	Root	[52,63,65]
*p*-Hydroxyphenylacetic acid	Leaf	[16,17]
Quercetin-3-*O*-galactoside	Leaf	[16,17,52,53,64,65]
	Stalk	[52,65]
	Root	[52,65]
Quercetin-3-O-rutinoside (Rutin)	Leaf	[16,17,52,53,63,65,66,67]
	Stalk	[52,63,65]
	Root	[52,63,65,66]
Apigenin-7-*O*-glucoside	Leaf	[16,17,52,53]
Quercetin	Leaf	[16,17,52,53,63,66,67]
	Stalk	[52,63]
	Root	[52,63,66]
Luteolin	Leaf	[16,17,53,64,67]
Naringin	Leaf	[16,17,53,63]
	Stalk	[63]
	Root	[63]
Kaempferol	Leaf	[16,17,52,53,63,64,67]
	Stalk	[52,63]
	Root	[52,63]
Apigenin	Leaf	[16,17,53,64,67]
Isorhamnetin-3-*O*-rutinoside	Leaf	[16,17,52,53,64,67]
	Stalk	[52]
	Root	[52]
Taxifolin	Leaf	[16,17]
Isorhamnetin-3-*O*-glucoside	Leaf	[17,52,63]
	Stalk	[52,63]
	Root	[52,63]
Daidzein	Leaf	[16,17]
Eriodictyol	Leaf	[16,17]
Chrysoeriol	Leaf	[16,17,53]
Chrysin	Leaf	[16,17]
Acacetin	Leaf	[16,17]
Genkwanin	Leaf	[16,17]
Galangin	Leaf	[16,17]
Kaempferide	Leaf	[16,17]
